# The role of ^m^SEPT9 in screening, diagnosis, and recurrence monitoring of colorectal cancer

**DOI:** 10.1186/s12885-019-5663-8

**Published:** 2019-05-14

**Authors:** Jie Sun, Fei Fei, Mingqing Zhang, Yuwei Li, Xipeng Zhang, Siwei Zhu, Shiwu Zhang

**Affiliations:** 1 Department of Pathology, Tianjin Union Medical Center, Tianjin, 300121 People’s Republic of China; 2 Nankai University School of Medicine, Nankai University, Tianjin, 300071 People’s Republic of China; 3 Department of colorectal surgery, Tianjin Union Medical Center, Tianjin, 300121 People’s Republic of China; 4 Tianjin Union Medical Center, Tianjin, 300121 People’s Republic of China

**Keywords:** Colorectal cancer, Septin 9, Microsatellite instability, CpG island methylator phenotype

## Abstract

**Background:**

The application of circulating, cell-free, methylated Septin9 (^m^SEPT9) DNA in screening and recurrence monitoring is highly promising. CpG island methylator phenotype (CIMP) is associated with microsatellite instability (MSI). The present study was performed to determine the diagnostic accuracy of ^m^SEPT9 for colorectal cancer (CRC) and to evaluate its utility in CRC screening and recurrence monitoring.

**Methods:**

For screening and diagnosis of CRC, peripheral ^m^SEPT9 detection and fecal occult blood test (FOBT) were performed in 650 subjects, then the level of CEA, CA19–9 and CA724 was quantified in 173 subjects. Clinicopathological parameters and mismatch repair protein were detected among subjects with CRC. For recurrence monitoring of CRC, the sensitivity of ^m^SEPT9 of 70 subjects was compared with tumor markers and contrast enhanced computed tomography (CECT).

**Results:**

Seventy-three percent of CRC patients were ^m^SEPT9-positive at 94.5% specificity, and 17.1% of patients with intestinal polyps and adenoma were ^m^SEPT9-positive at 94.5% specificity, which were higher than FOBT for the screening of CRC. The sensitivity and specificity of ^m^SEPT9 for diagnosis and recurrence monitoring were higher than that of CEA, CA19–9 and CA724. The combined detection of ^m^SEPT9 and CECT enhanced the sensitivity for recurrence monitoring. Pre-therapeutic levels of ^m^SEPT9 were strongly associated with TNM stage, Dukes stages and mismatch repair deficiency (dMMR).

**Conclusions:**

^m^SEPT9 analysis might be popularized as a routine biomarker for CRC screening. The combined detection of ^m^SEPT9 and CECT can play an important role for recurrence monitoring. CIMP was highly associated with the pathological stage of CRC and dMMR.

**Electronic supplementary material:**

The online version of this article (10.1186/s12885-019-5663-8) contains supplementary material, which is available to authorized users.

## Background

Colorectal cancer (CRC), a common malignant tumor of the digestive tract, presents with significant morbidity and mortality worldwide, and the age of onset tends to be low [1]. In China, especially in the first-tier cities, the incidence of CRC is increasing every year [2]. The main molecular mechanisms causing CRC include chromosome instability (CIN), microsatellite instability (MSI), and CpG island methylator phenotype (CIMP) [3]. Because colorectal cancer is highly occult in the initial stage, the key to early diagnosis, treatment, and improvement of prognosis lies in the discovery of key regulatory factors in the pathogenesis of CRC.

DNA methylation is an important epigenetic modification and CpG island is the main site of DNA methylation and is closely related to the occurrence and development of tumors [4]. Many abnormal gene methylations have been observed in colorectal cancer, and are closely related to CRC pathogenesis, prognosis and chemotherapy response [5, 6]. A study found that hypermethylation of the CpG island in the promoter region of the septin-9 gene, which acts as a tumor suppressor gene, inhibited the normal expression of the gene and consequent loss of its tumor suppressor function, thereby promoting the development of CRC [7]. Studies have shown that the rate of SEPT9 methylation in peripheral blood of patients with colorectal cancer with different clinicopathological features is different, and is positively correlated with the malignancy of CRC [8, 9]. After radical resection of colorectal cancer, the level of ^m^SEPT9 in peripheral blood decreased or became negative, but turned positive after recurrence, suggesting that ^m^SEPT9 in peripheral blood could be used for the pathological staging of colorectal cancer, and could be a molecular biological indicator for prognosis assessment, recurrence, and metastasis monitoring [10]. ^m^SEPT9 has superior sensitivity compared to fecal occult blood test (FOBT) and has better diagnostic biomarker complementary to FOBT as a screening tool for CRC [9], especially for CRC at early stages (I and II) [11].

The DNA repair system is closely involved in the pathogenesis of CRC. Decreased or lost function of mismatch repair in cells leads to MSI, and such MSI-containing genomes are unstable and have an increased susceptibility to tumors. Breakdown of mismatch repair system can lead to mutation and activation of oncogene [12]. CIMP-type CRC exhibits many molecular characteristics, including MSI, epigenetic silencing of mismatch repair gene MLH1, and TP53, BRAF and KRAS mutations. CIMP is also correlated with some clinicopathological features including tumor proximal localization, female patients, advanced age, mucinous tumors, and poorly differentiated tumors [13–15]. However, the prognostic value of CpG island methylation in CRC is still under study [16–19].

In this study, we compared the sensitivity and specificity of ^m^SEPT9 to FOBT, tumor markers, and contrast enhanced computed tomography (CECT) and analyzed the correlation of ^m^SEPT9 Cycle threshold (Ct) value with pathology characteristics and detected the expression of mismatch repair protein and analyzed its correlation with ^m^SEPT9 Ct value and CRC pathology. This study may provide some valuable information for the screening, diagnosis and recurrence monitoring of CRC, especially in those patients who are difficult to obtain biopsy specimens or are not willing to suffer intestinal preparation.

## Methods

### Ethics

This study was submitted to the ethics committee of Tianjin Union Medicine Center for review and approval prior to the start of the clinical study. All subjects provided an informed consent before blood collection.

### Subjects and study design

A total of 720 subjects including 600 cases of patients above 40 years old with high risk of CRC, 50 cases of preoperative patients who has been diagnosed with CRC and 70 cases of CRC patients after radical resection were recruited in this study to evaluate the suitability of ^m^SEPT9 DNA measurement in plasma for screening and diagnosis of CRC. The characteristics of subjects were listed in Table 1. High-risk of CRC were defined as follows: at least one first-degree relative with CRC; having a history of intestinal adenoma or polyps; FOBT positive; having two or more of the following at the same time: chronic constipation, chronic diarrhea, mucus and bloody stool, history of adverse life events (such as divorce, death of relatives, etc.), history of chronic appendicitis or appendectomy, and history of chronic cholecystitis or cholecystectomy [20–23]. These subjects were diagnosed by colonoscopy and subsequent pathological examinations. Subjects were then divided into the following clinical status groups: individuals suffering from CRC, adenoma or proliferative polyps, non-CRC gastrointestinal diseases (including inflammatory bowel diseases, colitis, ulcer, abscess, etc.), non-CRC cancers, and those having no evidence of diseases (NEDs). Then we analyzed the correlation between ^m^SEPT9 and clinicopathological characters. Seventy patients (1–3 year after surgery) with radical resection (stageI-III) were used to study the feasibility of measurement of ^m^SEPT9 DNA in plasma for recurrence monitoring, and the comparison with tumor markers and CECT. The final diagnosis of recurrence was drawn according to the results of colonoscopy and postoperative pathological examination. In this study, the diagnosis of recurrence includes local recurrence and distant metastasis.

**Table 1:** Characteristics of patients with CRC

All subjects	Number
600 subjects with and without high risk of CRC	600
Age	
41–50 years	81
51–60 years	162
61–70 years	229
> 70 years	128
Gender	
Female	315
Male	285
Smoking and drinking habits	
Non-smokers	208
Smokers	392
Non-alcoholic	151
Alcoholic	449
History and conditions	
One first-degree relative with CRC	72
Intestinal adenoma or polyps	56
FOBT positive	46
Chronic constipation	39
Chronic diarrhea	18
Inflammatory colon diseases	7
Mucus and bloody stool	12
Chronic appendicitis or appendectomy	6
Chronic cholecystitis or cholecystectomy	4
Adverse life events	12
Non	337
50 subjects with CRC before treatment	50
Age	
41–50 years	5
51–60 years	12
61–70 years	26
> 70 years	7
Gender	
Female	20
Male	30
70 subjects with CRC after radical resection	70
Age	
≤40 years	1
41–50 years	10
51–60 years	19
61–70 years	26
> 70 years	14
Gender	
Female	29
Male	41
Postoperative time	
6 months after surgery	17
12 months after surgery	19
24 months after surgery	20
36 months after surgery	14
Total	720

### Sample collection and storage

10 ml peripheral blood sample was collected in 10 ml tubes containing the K_2_EDTA anticoagulant (BD biosciences, NJ, USA). Plasma samples (3.5 ml) without apparent hemolysis, high bilirubin, chylemia, or visible particles or pellets were collected upon centrifugation and stored under − 20 °C within 2 weeks from the sample collection date. Blood samples of subjects with high risk of CRC were collected before colonoscopy examination and stored according to the above instructions. Blood samples of CRC patients who had taken colonoscopy examination were collected before surgery.

### ^m^SEPT9 methylation quantification

An improved SEPT9 gene methylation assay (Epigenomics AG for Epi proColon 2.0) was used for CRC detection in our study, in which the main improvements included a reduced number of PCR reactions and an increased throughput per run compared to the original reaction [24]. DNA was extracted from the plasma samples using the plasma processing kit manufactured by BioChain Science and Technology, Inc. (Beijing). The DNA was then incubated with bisulfite, during which unmethylated cytosine was converted to uracil, whereas methylated cytosines were not. Following this, the methylated target sequences in the bisulfite-converted DNA template were amplified by real-time PCR. PCR blocking oligonucleotides and methylation specific probes worked together to distinguish between methylated and non-methylated DNA. The sequences of primers, blockers, and probes for SEPT9 detection used in methylation-specific PCR amplification were as follows: forward primer, 5′-CCCACCAACCATCATAT-3′; Reverse primer, 5′-GTAGTAGTTAGTTTAGTATTTATTTT-3′; blocker, 5′-CATCATATCAAACCCCACAATCAACACACAAC-3′; probe1, 5′-GTTCGAAATGATTTTATTTAGTTGC-3′; probe2, 5′-CGTTGATCGCGGGGTTC-3′. PCR was performed in a 60 μL reaction system. The qPCR was performed in duplicate and the average value of Ct was calculated. 3.5 ml positive control contains 100 pg ^m^SEPT9 DNA, and 3.5 ml negative control contains 5 ng SEPT9 DNA. β-actin was used as the control to evaluate the plasma DNA quality and the validity of PCR amplification. The sequence of primers and probes for β-actin detection used in PCR amplification were as follows: forward primer, 5′-GTGATGGAGGAGGTTTAGTAAGTT-3′; reverse primer, 5′-CCAATAAAACCTACTCCTCCCTTAA-3′; and probe, 5′-ACCACCACCCAACACACAATAACAAACACA-3′. The thermocycling program was as follows: activation at 94 °C for 20 min; 45 cycles at 62 °C for 5 s, 55.5 °C for 35 s, and 93 °C for 30 s; and cooling at 40 °C for 5 s. The methylation of SEPT9 in plasma was measured by ABI7500 fluorescent PCR instrument. The Ct value of the control was less than or equal to 32.1. ^m^SEPT9 Ct cutoff value of 41 was established in this assay. If the Ct value was less than or equal to 41, the result was positive. If the Ct value was more than 41, the result was negative. The Ct value of ^m^SEPT9 is used as risk assessment for CRCs and The Ct value below 39 indicated the possibility of CRC in the patients.

### Fecal occult blood test (FOBT)

FOBT was performed one to 2 weeks before the detection of peripheral ^m^SEPT9, Immune colloidal gold technique was utilized for the detection of fecal occult blood and monoclonal antibodies were used to specifically target human hemoglobin in feces samples.

The reaction line (T) on cellulose nitrate membrane was coated with anti-HB1 monoclonal antibody and the control line (C) was coated with sheep anti-mouse polyclonal antibody. When detected, the human hemoglobin in the sample could bind to the colloidal gold-antibody coated at the front of the reagent to form an immune complex. As the chromatographic complex moves along the membrane band, if it is a positive sample, it can agglutinate to form a color band on the reaction line (T) and the control line (C), respectively. If it is a negative sample, it will only form a color band on the control line (C). The lowest detectable level of hemoglobin was 0.2 μg/ml, ranging from 0.2 μg/ml to 2000 μg/ml. For sample extraction, 10–50 mg sample was taken from 6 different parts of stool with stool bar and mixed well in 0.5 ml buffer solution for detection.

### CEA, CA 19–9 and CA724 quantification

The serum tumor markers carcinoembryonic antigen (CEA), carbohydrate antigen 19–9 (CA 19–9) and carbohydrate antigen 724 (CA724) were detected by electrochemiluminescence. The clinical significance about CEA, CA19–9 and CA724 were introduced in the Additional file 1. Positive values were defined using broadly accepted cut-offs (CEA: 0–5 ng/mL, CA19–9: 0–37 U/mL, CA724: 0–6.9 U/mL). Then we compared the sensitivity and specificity between ^m^SEPT9 and the tumor markers.

### Immunohistochemical staining

Sections of the CRC tissue sample were subjected to immunohistochemical analysis to detect the presence of mismatch repair proteins MLH1, MSH2, MSH6 and PMS2 and the expression of P53. The sections were deparaffinized, rehydrated, and boiled in citrate buffer (0.01 mmol/L, pH 6.0) for 20 min in a microwave oven. After the antigen retrieval, the sections were immersed in 3% H_2_O_2_ solution for 10 min to block endogenous peroxidase. The sections were blocked with 5% goat serum for 30 min at room temperature, then incubated with primary antibody at 4 °C overnight. After three 5 min washes in TBS, the sections were incubated with HRP-labeled secondary antibody for 2 h at room temperature. This was again followed by three 5 min washes in TBS. Diaminobenzidine-hydrogen peroxidase-chromogen-substrate system was used for signal conversion. Finally, hematoxylin co-staining was performed.

### Statistical analyses

ANOVA, Spearman’s rank correlations, t tests were performed to compare ^m^SEPT9 levels among different groups. The area under the curve (AUC) of the receiver operating characteristic curve (ROC) was computed to compare the differences among ^m^SEPT9 and glycoprotein tumor markers. Fisher exact test was used for univariate analysis, and Logistic regression analysis was used for multivariate analysis to study the relationships among clinical parameters, ^m^SEPT9 level and Dukes stages. Chi-square tests were used to estimate and test the association between mismatch repair (MMR) status and ^m^SEPT9 status. Two-sided *P* values of 0.05 were considered to be statistically significant.

## Results

### Utility of ^m^SEPT9 DNA in plasma for screening, diagnosis, and recurrence monitoring

To evaluate the utility of ^m^SEPT9 DNA in plasma for screening and diagnostic purposes, 600 subjects with high risk of CRC and 50 preoperative patients with CRC were enrolled in the current study. The subjects were grouped based on colonoscopy results and pathological diagnosis. A cycle threshold (Ct) cutoff value of 41 was established based on the training and testing study of BioChain Science and Technology, Inc. (Beijing). Table 2 lists the number of cases in each group and the corresponding positive detection rate. Thirteen patients were diagnosed with CRCs among 600 subjects based on the results of colonoscopy and pathological examination, so there were total 63 CRC patients in the analysis of ^m^SEPT9 for screening and diagnosis of CRC. The overall sensitivity for CRC detection was 73.0% (Table 3), and the positive detection rate in CRC group was increasing with the developing of pathological stage. However, the Dukes B stage has the biggest positive detection rate in our study which might related that mismatch repair deficiency (dMMR) mainly occurred in the Dukes B stage. The specificity was 94.5%, because the positive detection rate for NED subjects was only 5.5% (Table 2). The positive prediction values (PPV) and negative prediction values (NPV) for CRC were 63.0 and 96.5% respectively. However, the detection rates for adenoma and proliferative polyps, and non-CRC GI diseases were low (17.1 and 18.2%, respectively), which was not significantly different from the positive detection rate of the NED group, and therefore, has no diagnostic significance. Table 3 also shows that the sensitivity, specificity, PPV and NPV of ^m^SEPT9 was higher than FOBT, which indicated that the improved ^m^SEPT9 assay was specific for CRC detection, and better than FOBT for screening of CRC.

**Table 2:** Number of subjects enrolled as per diagnosis group and the positive detection rate for each group

Diagnosis group	Number	Positive detection rate[%(*n/N*)]
CRC		
Total	63	73.0% (46/63)
None	6	33.3% (2/6)
Dukes A	3	33.3% (1/3)
Dukes B	24	91.7% (22/24)
Dukes C	22	63.6% (14/22)
Dukes D	8	87.5% (7/8)
Adenoma and Polyps	82	17.1% (14/82)
Non-CRC GI diseases	11	18.2% (2/11)
Non-CRC GI cancers	0	NA
NED	494	5.5% (27/494)
Total	650

**Table 3:** Mean Ct value, Sensitivity, Specificity, PPV, and NPV of mSEPT9 and FOBT in the CRC group and adenoma and polyps’ group

	CRC		Adenoma and Polyps	
	^m^SEPT9	FOBT	^m^SEPT9	FOBT
Mean Ct value	39.24		42.65	
Sensitivity	73.0% (60.1%~ 83.1%)	58.7% (45.6–70.8%)	17.1% (10.0–27.3%)	12.2% (6.3–21.7%)
Specificity	94.5% (92.0%~ 96.3%)	91.9% (89.0–94.1%)	94.5% (92.0%~ 96.3%)	91.9% (89.0–94.1%)
PPV	63.0% (50.9%~ 73.8%)	48.1% (36.6–59.7%)	34.1% (20.6%~ 50.7%)	20.0% (10.5–34.1%)
NPV	96.5% (94.3%~ 97.9%)	94.6% (92.1–96.4%)	87.3% (84.1%~ 89.9%)	86.3% (83.0–89.1%)

**Fig. 1:**
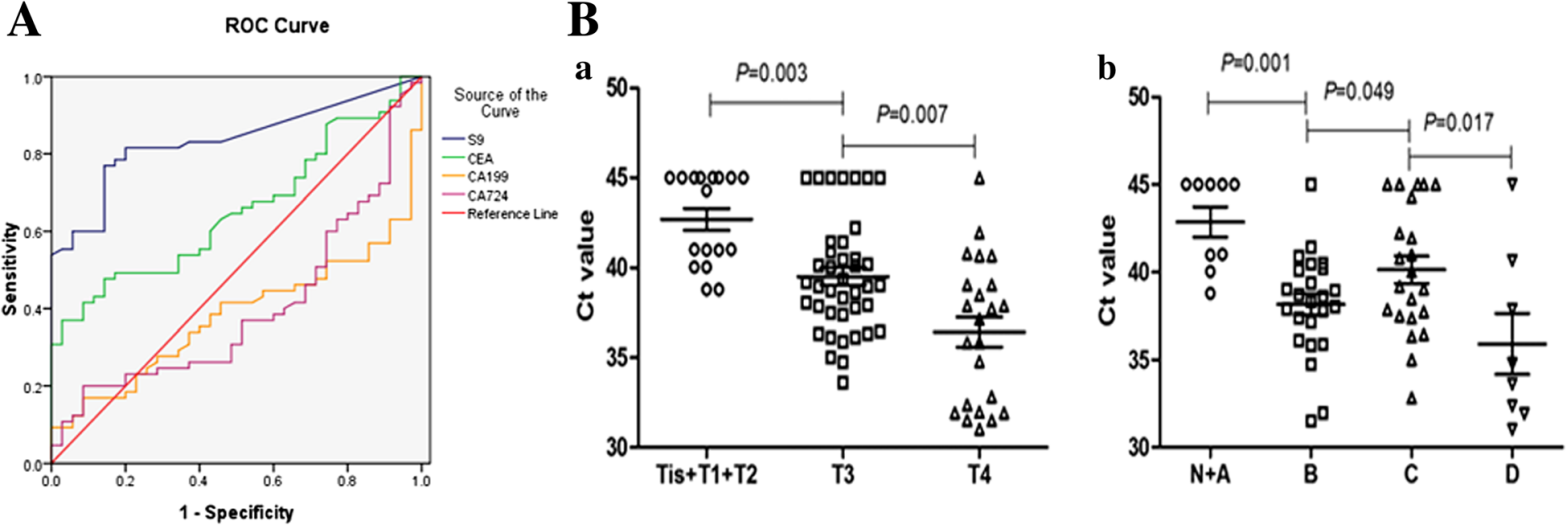
A. The ROC curve of ^m^SEPT9 (S9), CEA, CA19–9 and CA724. B. Stage-dependent Ct value of pre-therapeutic ^m^SEPT9 DNA in plasma. a) The Ct value of peripheral ^m^SEPT9 in each primary tumor (T) category. b) The Ct value of peripheral ^m^SEPT9 in each Dukes stage

The area under the ROC curve for CRC was calculated to be 0.835 (95% Confidence interval, 95%CI (0.758–0.913)) (Fig. 1A, Table 4), suggesting a high sensitivity and specificity of the assay in distinguishing CRC from NED subjects. Table 3 shows the mean Ct value of CRC group to be 39.24, and the mean Ct value of the group of patients suffering from adenoma and polyps to be 42.65, which indicated that when the Ct value of subject is lower than 39, the subject may have CRC. Thus, the Ct value of peripheral ^m^SEPT9 may be an effective diagnostic tool for CRC. To compare the sensitivity and specificity between ^m^SEPT9 and common tumor markers during the auxiliary diagnosis of CRC, pre-therapeutic CEA, CA 19–9 and CA724, serum levels were tested in 63 CRC patients and 60 subjects in NED group. Calculation of the area under the ROC curve for CRC showed the area of ^m^SEPT9 to be larger than that of CEA, CA19–9 and CA724 (Fig. 1A, Table 4). These data clearly show that the ^m^SEPT9 assay alone can detect CRC with high sensitivity.

**Table 4:** Area under the ROC

Area Under the Curve					
Test Result Variable(s)				Asymptotic 95% Confidence Interval	
	Area	Std. Error	Asymptotic Sig.	Lower Bound	Upper Bound
^m^SEPT9	0.835	0.040	0.000	0.758	0.913
CEA	0.654	0.054	0.012	0.548	0.759
CA19–9	0.381	0.055	0.051	0.274	0.489
CA724	0.400	0.058	0.099	0.285	0.514

To study the role of ^m^SEPT9 in recurrence monitoring, 70 patients with radical resection were recruited to detected peripheral ^m^SEPT9, tumor markers and CECT. After colonoscopy or postoperative pathological examination, there were 21 cases of recurrence, including 12 cases of local recurrence and 9 cases of distant metastasis. Table 5 shows the sensitivity and specificity of ^m^SEPT9 to be 71.4 and 98.0% respectively, and the PPV and NPV to be 93.8 and 88.9% for the recurrence of CRC, which was higher than tumor markers, but was lower than CECT, but the positive detection rate of combination of ^m^SEPT9 with CECT was higher than single CECT and the combination of tumor markers with CECT (Table 6), which showed that single mSEPT9 detection or the combination of ^m^SEPT9 and CECT were helpful for monitoring the recurrence of CRC.

**Table 5:** Sensitivity, Specificity, PPV, and NPV of ^m^SEPT9, tumor markers and CECT for recurrence

	Sensitivity	Specificity	PPV	NPV
^m^SEPT9	71.4% (47.7%~ 87.8%)	98.0% (87.8%~ 99.9%)	93.8% (67.7%~ 99.7%)	88.9% (76.7%~ 95.4%)
CEA	52.4% (30.3–73.6%)	91.8% (79.5%~ 97.4%)	73.3% (44.8%~ 91.1%)	81.8% (68.6%~ 90.5%)
CA19–9	33.3% (15.5–56.9%)	89.9% (77.0–96.2%)	58.3% (28.6–83.5%)	75.9% (62.5–85.7%)
CA724	42.9% (22.6–65.6%)	93.9% (82.1–98.4%)	75.0% (42.8–93.3%)	79.3% (66.3–88.4%)
CECT	85.7% (62.6–96.2%)	98.0% (87.8%~ 99.9%)	94.7% (71.9–99.7%)	94.1% (82.8–98.5%)

**Table 6:** The positive detection rate of different tests for recurrence

Tests and combinations	Positive detection rate [%(*n/N*)]
^m^SEPT9	71.4% (15/21)
CECT	85.7% (18/21)
^m^SEPT9 + CECT	95.2% (20/21)
CEA + CA199 + CA724	61.9% (13/21)
CEA + CA199 + CA724 + CECT	85.7% (18/21)

### Correlation between the level of pre-therapeutic ^m^SEPT9 DNA in plasma and pathological characteristics of CRC

Clinicopathologic information of 63 CRC patients was collected to study the specific correlation between peripheral methylation of SEPT9 and pathological manifestation. Detailed clinicopathologic parameters were summarized in Table 7. The mean Ct value of ^m^SEPT9 prior to surgery was significantly associated with TNM categories, Dukes stages, and gross tumor volume but not with tumor localization. The mean Ct value of ^m^SEPT9 was stage-dependent and showed a stepwise decrease according to local tumor stages (Tis-T4) (Fig. 1B –a) and Dukes stages (A-D) (Fig. 1B-b). However, the mean Ct value of Dukes B stage was higher than that of Dukes C stage, which may be related to dMMR. We also analyzed the relationships among clinical parameters, the value of ^m^SEPT9 and Dukes stages, and confirmed that TNM stage, tumor differentiation and ^m^SEPT9 level were related to the Dukes stages (Table 8). These results showed that the value of pre-therapeutic ^m^SEPT9 may be a significant tool for the pathological diagnosis of CRC, especially for the discrimination between localized and metastatic cases.

**Table 7:** Correlation between ^m^SEPT9 and pathological characteristics of CRC

Clinicopathological parameters	Number	Mean Ct value	*P* value
CRC cases	63(100%)		
Localization			
Colon	24(38.1%)	39.16	
Rectosigmoid transition	7(11.1%)	39.98	
Rectum	32(50.8%)	37.09	
Primary tumor (T)			
Tis	6(9.5%)	43.14	
T1	1(1.6%)	40.99	
T2	4(6.3%)	42.83	
T3	35(55.6%)	39.43	0.003
T4	17(27.0%)	36.53	0.007
Regional node (N)			
Nx	12(19.0%)	39.32	
N0	27(42.9%)	38.63	
N1	13(20.6%)	40.64	
N2	11(17.5%)	39.01	
Distant metastasis (M)			
Mx	55(87.3%)	39.83	
M0	0(0%)		
M1	8(12.7%)	35.91	0.009
Dukes stage			
None	6(9.5%)	43.14	
Dukes A	3(4.8%)	42.34	
Dukes B	24(38.1%)	38.16	0.001
Dukes C	22(34.9%)	40.14	0.049
Dukes D	8(12.7%)	35.91	0.017
Gross tumor volume			
None	8(12.7%)	37.06	
0–10 cm^3^	23(36.5%)	41.29	
> 10 cm^3^	32(50.8%)	38.31	0.002

**Table 8:** Correlation of clinical parameters and ^m^SEPT9 with Dukes stages

Clinicopathological parameters	Dukes A(n)	Dukes B(n)	Dukes C(n)	Dukes D(n)	*P*	OR (95%CI)
Gender					0.973	0.58(−3.669–6.557)
Female	1	10	10	4		
Male	2	14	12	4		
Age					0.744	0.459(−10.312–4.655)
≤ 60	0	9	9	3		
> 60	3	15	13	5		
Localization					0.405	0.338(−2.644–7.702)
Colon and Rectosigmoid transition	2	10	10	6		
Rectum	1	14	12	2		
Depth of invasion					0	0.104(−1.47–15.66)
T1 + T2	3	0	2	0		
T3	0	20	14	1		
T4	0	4	6	7		
Lymphatic metastasis (N)					0	0.042(0.744–39.596)
Nx + N0	3	24	1	0		
N1 + N2	0	0	21	8		
Distant metastasis (M)					0	0.166(−8.463–49.171)
Mx + M0	3	24	22	0		
M1	0	0	0	8		
Tumor differentiation					0.026	0.21(−16.733–3.676)
Well and moderately differentiated	3	18	11	2		
Poorly differentiated	0	6	11	6		
Gross tumor volume					0.282	0.69(−11.985–7.936)
0–10 cm^3^	2	6	10	2		
> 10 cm^3^	1	18	12	6		
^m^SEPT9					0.026	0.766(−13.507–9.948)
Positive	1	22	14	7		
Negative	2	2	8	1		

### The possible correlation between SEPT9 hypermethylation and dMMR

Since the mean Ct value of Dukes B was higher than that of Dukes C, we further studied the potential mechanism contributing to this observation. It has been shown that the MSI mainly occurred in the Dukes B stage, and hence we detected the expression of mismatch repair protein related to MSI-H and analyzed the correlation between dMMR and ^m^SEPT9. We found that the mean Ct value of the dMMR group was higher than that of the MMR-proficient (pMMR) group, and the positive detection rate was also higher than that of the pMMR group. In addition, the mean Ct value and positive detection rate of Dukes B stage in the dMMR group was higher than that in the pMMR group (Table 9), indicating that dMMR might enhance the hypermethylation of SEPT9.

**Table 9:** The difference of mean Ct value of mSEPT9 between dMMR and pMMR

	Mean Ct value	Positive detection rate	Proportion at Dukes B stage	Mean Ct value at Dukes B stage	Positive detection rate at Dukes B stage
dMMR	38.65	80%	55%	37.05	100%
pMMR	39.59	74.2%	42%	39.10	84.6%

## Discussion

CRC is caused by the gradual accumulation and interaction of pathogenic mechanisms such as polygenic mutation and epigenetic changes, and hence, studying the correlation between multiple pathogenesis of CRC is of great clinical significance, in order to explore simple, safe, more specific, and sensitive molecular indicators for screening, diagnosis, and prognosis evaluation of the disease. In a clinical setting, the screening of CRC at an early stage is still the most effective way to reduce morbidity and mortality [25]. The sensitivity and specificity of common CRC screening tests, such as FOBT and glycoprotein tumor marker CEA measurement, are low. However, patients are always easy to accept these screening methods because of the non-invasion [26]. Our study showed that FOBT demonstrated low sensitivity towards early screening of CRC. Although invasive colonoscopy has the highest sensitivity and specificity for CRC and adenoma detection, it has the lowest patient compliance rate due to the need of bowel preparation and discomfort during the test. Furthermore, some patients with severe cardiopulmonary insufficiency, enterostenosis or intestinal perforation are not suitable for invasive test. Hence, it is important to adopt a simple method with high sensitivity to make up for the limitations of the above-mentioned common detection methods.

Aberrant epigenetic modifications are an early event in carcinogenesis, with the epigenetic landscape continuing to change during tumor progression and metastasis. Due to the stability of cell-free DNA (cfDNA), the abnormal level of methylated DNA has been regarded as a promising candidate for a cancer biomarker [27]. Nevertheless, it still posed a great challenge for the early screening of CRC due to the lack of tumor markers with high sensitivity and specificity [28]. Among the methylated genes in CRC, epigenetically modified ^m^SEPT9 has been highlighted as an ideal candidate biomarker [29]. The circulating ^m^SEPT9 in plasma is derived from apoptotic cells shed from the tumor [30]. A meta-analysis showed that ^m^SEPT9 could be used to diagnose CRC in healthy individuals under the 2/3 algorithm [9, 31]. In this study, CRC was screened for using the ^m^SEPT9 assay and the positive detection rate, sensitivity, specificity, PPV, and NPV were analyzed. The results showed good sensitivity towards CRC detection and exhibited a high specificity due to low false-positive rate in adenoma, proliferative polyps, non-CRC GI diseases, and non-CRC cancer detections. Thus, quantification of the peripheral ^m^SEPT9 appears to be a simpler, cheaper, and more efficient tool for CRC screening. In addition, our study showed that 71.4% of the CRC patients with recurrence were ^m^SEPT9-positive at 98.0% specificity, which was better than that exhibited by the glycoprotein tumor marker. What’s more, the positive detection rate of combination of ^m^SEPT9 with CECT was higher than signal CECT and was higher than the combination of tumor marker with CECT. Thus, a validated blood-based biomarker like ^m^SEPT9 for CRC may help to identify patients with radiologically undetectable recurrence or metastases [32].

In CRC, hypermethylation of the gene in the promoter region is associated with transcriptional activation, leading to decreased expression of tumor suppressor genes and DNA repair genes, which affect the normal function of cell apoptosis, DNA repair and cell cycle regulation. The degree of methylation of Septin9 gene is accompanied by the development of colorectal tumors, and appears in the early stage of CRC without obvious tissue changes. The degree of methylation of Septin9 gene is gradually increased with the development of pathological tissues [33]. The level of ^m^SEPT9 associated with the clinicopathologic characteristics. Fu et al. reported that CRC cases with tumor size > 5 cm showed a significantly higher positive rate of ^m^SEPT9 than those with tumor size ≤5 cm, which was similar to our study. They also found that CRCs with higher histological grade showed a higher positive rate of ^m^SEPT9 [34]. Previous study by Xie et al. showed that ^m^SEPT9 had higher sensitivity for patients with distant metastasis [35]. Methylation levels of SEPT9 were significantly associated with nodal (N), tumor (T) and metastasis (M) categories, as well as Dukes category, which indicated that peripheral ^m^SEPT9 in plasma could be a powerful auxiliary molecular staging parameter, and together with TNM classification, facilitate molecular disease staging of CRC.

CIMP with multiple promoter methylated loci has been observed in a subset of CRC cases. CIMP status, which is closely associated with specific clinicopathological and molecular characteristics, is considered a potential predictive biomarker for efficacy of cancer diagnosis and treatment [36]. CIMP status in CRC has been shown to be associated with some specific clinical features (female sex, older age, family history of CRC, proximal location in the colon, mucinous cell differentiation) as well as some genetic features (sporadic MSI, wild-type TP53, mutations of BRAF and KRAS, and MLH1 promoter methylation) [37–39].The MSI is caused by a hypermutable phenotype due to loss of DNA mismatch repair mechanisms, which has large proportion in stage II of CRC. In our study, maximum proportion of dMMR is observed in the Dukes B stage. dMMR could be induced by gene promoter hypermethylation or germinal mutations. The MMR genes, including MLH1, MSH2, MSH6, PMS1 and PMS2, could easily mutate in CRCs with MSI [40]. In our study, the Ct value of CRC patients with dMMR was lower than the patients with pMMR, which indicated that dMMR might promote the methylation of SEPT9.

## Conclusions

Our study indicated that peripheral ^m^SEPT9 may be useful for the screening, early diagnosis, and recurrence monitoring of CRC, and related to dMMR. However, there are several limitations in our study, such as the numerous heterogeneities and small sample numbers of some subgroups, which needs to be improved in the future. Therefore, we intend to expand the sample size to further study the interaction between epigenetics and genetics, and the molecular typing of CRC, with an aim to improve the quality of individualized clinical treatment.

## Additional file

Additional file 1: The clinical significances about CEA, CA19–9 and CA724. The clinical significances of CEA, CA19–9 and CA724 in CRCs. (DOCX 12 kb)

## Abbreviations

CA 19–9: Carbohydrate antigen 19–9
CA724: Carbohydrate antigen 724
CEA: Carcinoembryonic antigen
CECT: Contrast enhanced computed tomography
cfDNA: cell-free DNA
CIMP: CpG island methylator phenotype
CIN: Chromosome instability
CRC: Colorectal cancer
Ct: Cycle threshold
dMMR: Mismatch repair deficiency
FOBT: Fecal occult blood test
mSEPT9: Methylated Septin9
MSI: Microsatellite instability
NPV: Negative prediction values
PPV: Positive prediction values
