# Supplementary material for: The role of mSEPT9 in screening, diagnosis, and recurrence monitoring of colorectal cancer
Source: BMC Cancer. 2019 May 14;19:450. doi: 10.1186/s12885-019-5663-8 (PMC6518628; doi:10.1186/s12885-019-5663-8)
Supplement: Supplementary file 1 — The clinical significances about CEA, CA19–9 and CA724. The clinical significances of CEA, CA19–9 and CA724 in CRCs. (DOCX 12 kb) [file 12885_2019_5663_MOESM1_ESM.docx]

**The clinical significances about CEA, CA19-9 and CA724:**

CEA measurement of patients with colorectal cancer before surgery is recommended by the American Society of Clinical Oncology (ASCO) due to the complementary diagnosis in pathologic staging and surveillance in recurrence. CA19-9 is a mucin-type glycoprotein tumor marker, which is glycolipid on cell membrane. CA724 belongs to mucin carcinoembryonic antigen. They are both gastrointestinal tumor associated antigen.
